# Complex Analysis of Urate Transporters *SLC2A9, SLC22A12* and Functional Characterization of Non-Synonymous Allelic Variants of GLUT9 in the Czech Population: No Evidence of Effect on Hyperuricemia and Gout

**DOI:** 10.1371/journal.pone.0107902

**Published:** 2014-09-30

**Authors:** Olha Hurba, Andrea Mancikova, Vladimir Krylov, Marketa Pavlikova, Karel Pavelka, Blanka Stibůrková

**Affiliations:** 1 Institute of Inherited Metabolic Disorders, First Faculty of Medicine, Charles University in Prague and General University Hospital in Prague, Prague, Czech Republic; 2 Charles University in Prague, Faculty of Science, Department of Cell Biology, Prague, Czech Republic; 3 Institute of Rheumatology, Prague, Czech Republic; University of Tokushima, Japan

## Abstract

**Objective:**

Using European descent Czech populations, we performed a study of *SLC2A9* and *SLC22A12* genes previously identified as being associated with serum uric acid concentrations and gout. This is the first study of the impact of non-synonymous allelic variants on the function of GLUT9 except for patients suffering from renal hypouricemia type 2.

**Methods:**

The cohort consisted of 250 individuals (150 controls, 54 nonspecific hyperuricemics and 46 primary gout and/or hyperuricemia subjects). We analyzed 13 exons of *SLC2A9* (GLUT9 variant 1 and GLUT9 variant 2) and 10 exons of *SLC22A12* by PCR amplification and sequenced directly. Allelic variants were prepared and their urate uptake and subcellular localization were studied by *Xenopus* oocytes expression system. The functional studies were analyzed using the non-parametric Wilcoxon and Kruskall-Wallis tests; the association study used the Fisher exact test and linear regression approach.

**Results:**

We identified a total of 52 sequence variants (12 unpublished). Eight non-synonymous allelic variants were found only in *SLC2A9*: rs6820230, rs2276961, rs144196049, rs112404957, rs73225891, rs16890979, rs3733591 and rs2280205. None of these variants showed any significant difference in the expression of GLUT9 and in urate transport. In the association study, eight variants showed a possible association with hyperuricemia. However, seven of these were in introns and the one exon located variant, rs7932775, did not show a statistically significant association with serum uric acid concentration.

**Conclusion:**

Our results did not confirm any effect of *SLC22A12* and *SLC2A9* variants on serum uric acid concentration. Our complex approach using association analysis together with functional and immunohistochemical characterization of non-synonymous allelic variants did not show any influence on expression, subcellular localization and urate uptake of GLUT9.

## Introduction

The correlation between increased serum uric acid level (hyperuricemia) and hypertension, cardiovascular disease, insulin resistance, metabolic syndrome and renal disorders has been recently described [1]–[3]. Hyperuricemia depends on the balance of endogenous production and the renal excretion of uric acid (UA). Population studies found the prevalence of the hyperuricemia in men at 24–29%, in women at 2.6–20% and rarely among children. The proportion of heritability of serum UA is 0.38–0.63 [4]–[7].

UA is the end product of purine metabolism in humans. The absence of hepatic enzyme uricase in humans (and great apes) and effective renal urate reabsorption contribute to tenfold higher blood urate levels in humans compared to other mammals. About 75% of daily production of UA is excreted by the kidney while 25% is eliminated by the gastrointestinal tract. Transport mechanisms for urate are localized in the proximal tubule where urate is secreted and extensively reabsorbed. As a result, the ratio of excreted urate is only approximately 10%.

Several proteins have been shown to transport urate in the proximal tubule on the apical and basolateral membranes. The major genes influencing the level of serum UA are *SLC2A9, SLC22A12* and *ABCG2* [8]–[10]. The *SLC22A12* gene maps to chromosome 11q13 and codes two transcript variants of the URAT1 transporter specifically expressed on the apical membrane of the proximal tubule of the human kidney (10 exons, 553 and 332 amino acids). *SLC2A9* gene coding glucose transporter GLUT9 was localized on chromosome 4p15.3–p16. It exists in two variants: GLUT9 variant 1 (12 exons, 540 amino acids) and GLUT9 variant 2 (13 exons, 512 amino acids). GLUT9 variant 1 is transcribed primarily in the liver, on the basolateral membrane of the proximal tubule, in leukocytes and the placenta. Expression of GLUT9 variant 2 was observed only on the apical membrane of the proximal tubule and placenta.

Results from a series of GWAS studies (genome-wide association scans) revealed a significant correlation between genetic variants in the gene *SLC2A9* and serum UA level, the excretion fraction of UA, gout and body mass index [9]–[11]. The importance of URAT1 and GLUT9 for regulating blood urate levels was confirmed by the finding of causal mutations in patients with idiopathic renal hypouricemia type 1 (OMIM #220150, RHUC1) and type 2 (OMIM #612076, RHUC2). To date, more than 100 patients with a loss-of-function mutation (compound heterozygous and/or homozygous) in the *SLC22A12* gene have been found with most of the described patients being Japanese [8], [12]; additionally, more than ten patients with RHUC, caused by heterozygous defects in the *SLC2A9* gene, coding for GLUT9, have also been described in Japan (OMIM #612076, RHUC2) [13], [14]. Homozygous and/or compound heterozygous loss-of-function mutations in *SLC2A9*, responsible for severe hypouricemia (often complicated by nephrolithiasis and AKI), have also been reported [15]–[17]. Recent studies have suggested that RHUC is not necessarily restricted to East Asian populations, as previously thought [18].

Even with much research into renal uric acid transport, a complete understanding of the regulation of renal uric acid handling has not yet been fully elucidated. OAT4 (*SLC22A11*) and OAT 10 (*SLC22A13*) have been linked to transport of UA on the apical membrane of the proximal tubules [19], [20]. The secretion part of the transport of UA principally ensures the production of highly a variable gene ATP-binding cassette, subfamily G, member 2 (*ABCG2/BCRP*) ABCG2. Allelic variant p.Q141K resulted in 53% reduced transport of UA and at least 10% of all gout cases of European descent are attributable to this causal variant [21], [22]. In two hyperuricemia patients, it was reported that mutations in the human sodium phosphate transporter 4 (hNPT4; SLC17A3) exhibited a reduced urate efflux [23]. The other transporter is the renal sodium-dependent phosphate transport protein 1 (*SLC17A1*) which functions as a urate secretor and an SNP analysis in patients with gout identified a significant association with reduced levels of serum UA [24], [25]. Additionally, OAT1 (*SLC22A6*), OAT3 (*SLC22A8*) [26] and the multidrug resistance protein 4, a member of the MRP/ABCC subfamily of ATP-binding cassette transporters, work as urate secretors [27].

These findings indicate that serum UA concentrations are largely determined by the relative balance between urate absorption and secretion across the proximal tubule. Moreover, these data suggest a new concept of renal UA transport – a multimolecular complex "transportosome" that probably involves cooperation between multiple transporters [28]. The impact of common non-synonymous allelic variants on the function of GLUT9 and URAT1 has not been reported to date. The aim of our study is to clarify the frequency, the role and the function effect of individual allelic variants of urate transporters *SLC2A9* and *SLC22A12* to the serum level of UA in a group of 250 Czech individuals (150 controls, 54 nonspecific hyperuricemics and 46 primary gout and/or primary asymptomatic hyperuricemia patients). To this purpose, we have: (1) genotyped polymorphisms in the *SLC2A9* and *SLC22A12* genes; (2) functionally characterized found non-synonymous allelic variants and (3) performed a case-control genetic association study on both exon and intron allelic variants.

## Materials and Methods

### Subjects

The analyzed set consists of two groups: a normouricemic group of 150 subjects and a hyperuricemic group of 100 subjects. The normouricemic group was selected from the set of 591 controls already biochemically and clinically characterized from a previous study [29]. Normouricemia was defined as serum UA concentration greater or equal to 120 µmol/L and less or equal to 416 µmol/L for men or 340 µmol/L for women, respectively. Prior to selection, we excluded individuals using drugs that influence the serum UA concentrations (mostly hormonal contraception and substitute hormonal therapy, acetylsalisyl acid and others). In the random selection of 150 individuals for detailed genomic analysis, we accented the younger and older age category as follows: 20 men and 20 women younger or aged 40 years, 35 men and 35 women aged between 40 and 55 years and 20 men and 20 women aged more than 55 years.

The hyperuricemia group of 100 individuals consists of two separate sets. The first part consists of 46 primary gout and/or primary asymptomatic hyperuricemia patients from the Institute of Rheumatology. The definition of hyperuricemia was as follows: >420 µmol/l at two repeated measurements at intervals of at least 4 weeks in men and >360 µmol/l at two repeated measurements at the same interval in women. Gouty arthritis was diagnosed according to the American college of rheumatology criteria, i.e. either in the presence of crystals sodium urate in polarized microscope in the synovial fluid or if subjects fulfil 6/12 clinical criteria [30]. Patients suffering from secondary gout and other purine metabolic disorders associated with pathological concentrations of serum UA (such as the defect of the hypoxanthine-guanine phosphoribosyl-transferase and phosphoribosylpyrophosphate synthetase 1) were excluded. This set is complemented with 39 hyperuricemic individuals from the same set (591 controls) as the normouricemic group, and 15 hyperuricemic individuals from the 297 cardiovascular patients of the same study. We excluded individuals using drugs that lower the serum UA concentrations. We included all hyperuricemic individuals fulfilling the selection criteria. The study was approved by the Ethics Committee of Charles University – First Faculty of Medicine (no. 146/09); all subjects gave their informed written consent.

### PCR amplification of SLC22A1, SLC2A9 and sequence analysis

The genomic DNA for PCR analysis was isolated from subjects' blood samples (Qiagen columns). 13 exons of *SLC2A9* (GLUT9 variant 1 and GLUT9 variant 2) and 10 exons of *SLC22A12* were amplified using PCR and sequenced directly. Primer sequences and PCR conditions are summarized in Table S2. Fifty nanograms of genomic DNA was amplified in 50 µl containing 2.5 U Taq-Purple DNA polymerase, 200 µM dNTPs and 0.15 µM primers. Amplification products were gel purified using 1% agarose gel in 1xTAE buffer and Wizard SV gel and Gel/PCR DNA Fragments Extraction Kit (Geneaid, Taiwan). DNA sequencing was performed with an automated DNA sequencer (Applied Biosystems 3100-Avant Genetic Analyzer; Applied Biosystems, USA).

### Functional studies

The non-synonymous allelic variants of GLUT9 were tested using *in vitro* expression analysis in the *Xenopus* oocyte. The protocol was approved by the Committee on the Ethics of Animal Experiments of the Faculty of Science, Charles University and all surgery was performed under tricaine by certified person regarding the approval against the cruelty on laboratory animals (no. CZU 303/99).

*SLC2A9* allelic variants (variant 1 and 2) were prepared from the wt (GeneBank NM_020041.2 and NM_001001290.1) employing a Gene Tailor Site-Directed Mutagenesis Kit (Invitrogen). Final products were cloned into pcDNA3 vector. Subsequently, capped cRNAs were synthetized using a T7 mMESSAGE mMACHINE kit (Ambion, Inc.). Preparation of *Xenopus laevis* oocytes for urate uptake studies was done as described previously [18]. Particular allelic variants were always represented by six groups (experimental repeats) each containing five oocytes (30 oocytes in total). 50 ng of corresponding cRNA was injected into each oocyte using a Narishige IM300 pneumatic microinjector. Uptake studies with radioactive labeled urate were performed according to Stiburkova et al. [18]. The subcellular localization of allelic variants was determined using immunocytochemical analysis. Immunodetection of GLUT9 was performed on three-micrometer paraffin sections using rabbit anti-*SLC2A9* polyclonal C-terminus antibody (MBL, Japan). Detection of bound primary antibody was achieved using Alexa Fluor 488-conjugated with anti-rabbit IgG (diluted 1∶500; Molecular Probes, Invitrogen, Paisley, UK). For image acquisition, a Leica TCS SP2 confocal microscope was used equipped with an AOBS (Acousto-Optical Beam Splitter). Green fluorescence was visualized using an argon 488 nm laser with an emission bandwidth of 500–550 nm. Autofluorescence was also acquired using a 405 nm excitation laser with a 410–470 nm emission bandwidth.

### Case-control study

The possible effect of allelic variants found in the 250 subjects of the study was analyzed through three separate analyses. First, we compared the genotype distribution in the normouricemic group with the genotype distribution in the hyperuricemia group (codominant model). Second, we compared the genotype distribution in the normouricemic group with the primary gout/primary hyperuricemia group. In both comparisons we tested the null hypothesis of no difference in genotype distribution against an alternative of difference of any kind. Third, we modeled the serum UA concentration for normouricemics with genotype as independent variable, considering gender, age, use of drugs lowering the serum UA level and anticoagulants, hypertension, fasting glycaemia and diabetes status as covariates. We have tested the null hypothesis of no effect of the respective allelic variant on serum UA concentration against the alternative of effect of any kind.

### Statistical analysis

The data were summarized as absolute and relative frequencies, medians and geometric means where appropriate. For the functional studies, we used the Wilcoxon-Mann-Whitney test and/or Kruskall-Wallis test to compare the measured amount of ^14^C urate uptake into oocytes expressed wild-type variant transporter and oocytes expressed the specific allelic variant. In the case-control study, we used the Fisher exact test to compare the genotype frequencies in the normouricemia and the hyperuricemia/primary gout groups. We modeled the logarithmically transformed serum uric acid concentrations using multivariable linear regression. For the Manhattan plot we used the -log_10_(p-value) of either the Fisher exact test for genotype distribution comparison or of the goodness-of-fit test in the linear regression model with and without the respective variant. Bonferroni correction was used to correct for multiple testing, with the overall level of statistical significance set to 0.05. The data were analyzed using the statistical language and environment R [31], version 3.0.2; the association study calculations and the graphs were performed using the R library SNPassoc.

## Results

### Subjects

The demographic and biochemical characteristics of the subjects are summarized in Table 1. While we could keep the gender- and age-balanced structure of the normouricemic group, we had problems in recruiting enough hyperuricemic subjects, especially women, and we included all available individuals fulfilling the selection criteria. The hyperuricemic group is thus not age and gender balanced. The fasting glycaemia, diabetes and hypertension data were available for all the normouricemic and 56 (56%) hyperuricemic individuals only. There are few normouricemic individuals with either clinically diagnosed hypertension or diabetes mellitus. There is a more important share of hypertonic individuals among the measured hyperuricemics, however, there might be a slight bias as those individuals were partly selected from the atherosclerotic group.

**Table 1:** Demographic, anamnestic and biochemical characteristics of the normouricemic and hyperuricemic groups.

		Normouricemic group			Hyperuricemic group			Primary gout/hyperuricemia subset		
	unit/category	men	women	total	men	women	total	men	women	total
Number of subjects	N	75	75	150	86	14	100	43	3	46
Primary gout	N	-	-	-	-	-	-	37	1	38
Primary hyperuricemia	N	-	-	-	-	-	-	12	2	14
Proportion of men/women		50%	50%	-	86%	14%	-	93%	7%	-
Age *	years	49	48	49	53.5	52	53	59	62	59.5
Age (category)	≤40	20	20	40	12	1	13	7	0	7
	>40 and ≤55	35	35	70	33	8	41	10	0	10
	>55	20	20	40	41	5	46	26	3	29
Serum uric acid*	µmol/l	306	222	-	435	366	-	392	449	-
Creatinine*	µmol/l	90	75	91	94	76.5	82	91	78	89
Hypertension (clinical)	N (proportion)	7 (9%)	1 (1%)	8 (5%)	16#	5#	21#	-	-	-
Diabetes mellitus	N (proportion)	2 (3%)	0	2 (1%)	4#	1#	5#	-	-	-
Fasting glycemia	mmol/l	5.27	4.86	5.03	5.37#	5.34#	5.36#	-	-	-
Allopurinol use	N (proportion)	0	0	0	31 (36%)	1 (7%)	32 (32%)	21 (49%)	1 (3%)	22 (48%)

Note: * data for age, serum uric acid concentrations, creatinine and fasting glycemia are presented as medians. The totals in serum uric acid concentrations are not presented because of their lack of meaning due to known gender-specific differences.

# The data on hypertension, diabetes mellitus and glycemia are known for only 56 individuals out of 100 in the hyperuricemic group.

### Analysis of SLC22A12 and SLC2A9

In the *SLC2A9* gene, 25 intronic sequence variants (nine variants unpublished) and 15 exon sequence variants (one synonymous variant unpublished) were detected. From these 15 exon sequence variants, there were eight non-synonymous: p.A17T (rs6820230) - only for GLUT9 variant 1, p.G25R (rs2276961) - only for GLUT9 variant 2, p.V169M (rs144196049), p.T275M (rs112404957), p.D281H (rs73225891), p.V282I (rs16890979), p.R294H (rs3733591) and p.P350L (rs2280205) and possibly changing the activity of GLUT9. In the *SLC22A12* gene, three variants in promoter region, four variants in intronic regions (two variants unpublished) and five synonymous exon variants were detected. All identified variants; their genotype distribution and mutant allele frequency in normouricemic and hyperuricemic group and in primary gout/hyperuricemia subgroup are shown in Table S1.

### Functional studies

**Figure 1:**
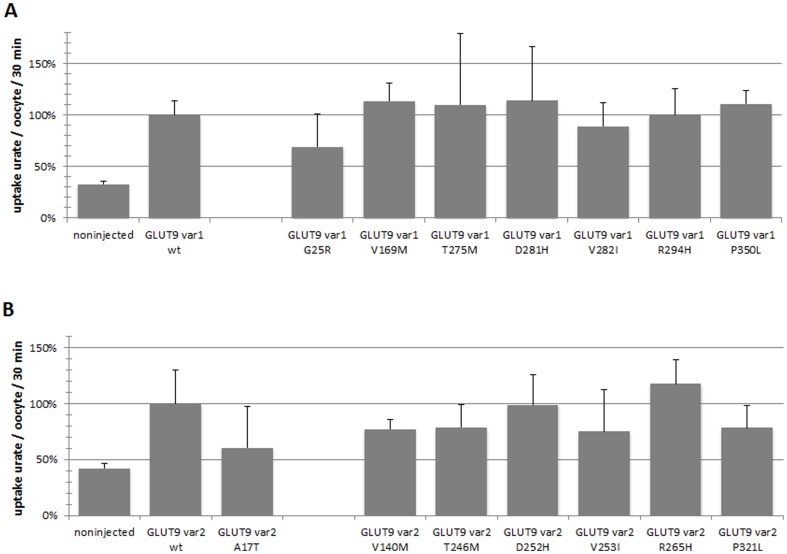
Urate uptake in oocytes. Transport activity is expressed as % urate uptake in oocytes injected with 50 ng of cRNA encoding the wild-type or the particular analyzed allelic variants. The uptake of 100 µM [^14^C]urate in oocytes was measured after 30 minutes incubation. Our experiments showed similar urate transport activity of GLUT9 variant 1 wt and GLUT9 variant 2 wt. GLUT9 wt variants were set to 100%. The average of six measurements each with five oocytes in group is shown and error bars represent standard error. (A) GLUT9 variant 1 (B) GLUT9 variant 2.

**Figure 2:**
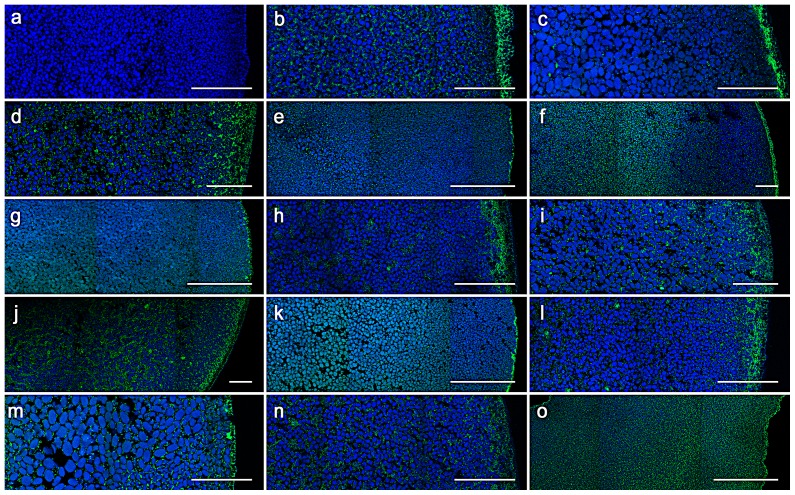
Immunocytochemical analysis of *Xenopus oocytes*. Immunocytochemical analysis of *Xenopus oocytes* injected with 50 ng of cRNA encoding the wild-type (wt) or mutant GLUT9 performed with rabbit anti-*SLC2A9* polyclonal antibody. The signal of protein is green while autofluorescent granules in cytoplasm of oocytes give a blue signal. (a) Non-injected oocytes, (b) oocytes injected with wt cRNA GLUT9 variant 1, (c) oocytes injected with wt cRNA GLUT9 variant 2, (d) GLUT9 variant 2 A17T, (e) GLUT9 variant 1 G25R, (f) GLUT9 variant 1 V169M, (g) GLUT9 variant 2 V140M, (h) GLUT9 variant 2 T246M, (i) GLUT9 variant 2 D252H, (j) GLUT9 variant 1 V282I, (k) GLUT9 variant 2 V253I, (l) GLUT9 variant 1 R294H, (m) GLUT9 variant 2 R265H, (n) GLUT9 variant 1 P350L, (o) GLUT9 variant 2 P321L. Scale bar represents 50 µm.

The eight non-synonymous allelic variants of GLUT9 were tested for urate transport activity in the Xenopus oocyte. As shown in Figure 1, the urate transport of GLUT9 variant 1 and GLUT9 variant 2 allelic variants were not significantly changed in comparison with the wt (wild type) variants, p<0.05. To investigate the expression and plasma membrane targeting of GLUT9 variant 1 and GLUT9 variant 2 allelic variants, sections of cRNA-injected oocytes stained with anti-GLUT9 polyclonal antibody were analyzed. Oocytes expressing the wt GLUT9 variant 1, wt GLUT9 variant 2 and all non-synonymous allelic variants exhibited continuous GLUT9 immunostaining on the plasma membrane and dispersed finely granular staining in the cytoplasm, Figure 2.

### Case-control study

**Figure 3:**
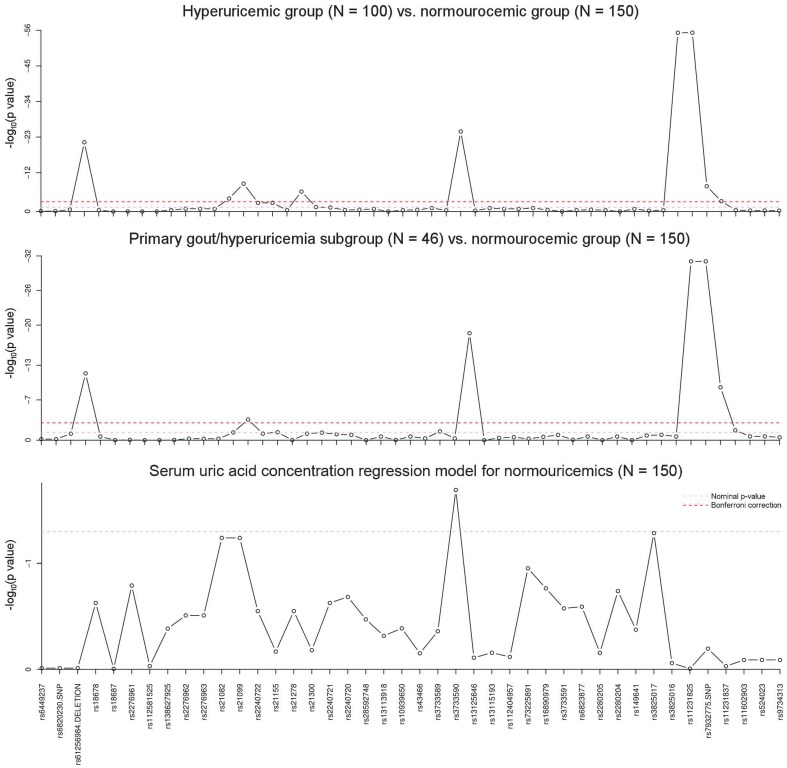
Plot of -log_10_(p-value) for the analysis of association of studied allele variants with hyperuricemia/primary gout and serum uric acid concentration. The dependent variable is the -log_10_(p-value) of either the Fisher exact test for genotype distribution comparison (top and middle figure) or the -log_10_(p-value) of the goodness-of-fit test in the linear regression model with and without the respective variant (bottom figure).

The results from the three association analyses are shown by the three Manhattan plots in Figure 3 and summarized in Table S2. The case-control comparison of the genotype distribution in the normouricemic and hyperuricemic groups detected eight candidate alleles out of 52 (rs18678, rs2240722, rs21155, rs2240720, rs4292327, rs8359, rs8361, rs7932775) that had significantly different genotype distributions (p-values <0.0009 = 0.05/52). Four of them (rs18678, rs4292327, rs8359 and rs8361) have extremely low p-values. This is due to the fact that these intronic variants showed only wild type allele in either the normouricemic (rs4292327, rs8359 and rs8361) or in the hyperuricemic (rs18678) groups while the mutant allele was quite frequent in the other comparison group. Six of the previously found candidates (rs18678, rs21155, rs4292327, rs8359, rs8361, rs7932775) were statistically significant in the case-control comparison of the primary gout/hyperuricemia group with the normouricemic group (p-values <0.0009 = 0.05/52). All but one (rs7932775) of these SNPs are located in the intron regions of the *SLC2A9* and *SLC22A12* genes. Genotype distribution, variant allele frequency and geometric means of serum UA concentration for the candidate allelic variants are presented in Table 2. The regression analysis of the serum UA concentrations in normouricemics, controlled for the gender, age, using of drugs lowering the serum UA level and anticoagulants, hypertension, fasting glycaemia and diabetes mellitus, did not find any variants that would have shown any statistically significant effect on serum UA concentrations.

**Table 2:** Genotype distribution, variant allele frequency and geometric mean of serum uric acid concentrations (µmol/l) for allelic variants that showed possible association with hyperuricemia (W wild type, M mutant).

	Normouricemic group - men (N = 75)								Normouricemic group - women (N = 75)							
significantly associated allelic variants	genotype distribution (N)			allele frequency %		geometric mean of serum UA			genotype distribution (N)			allele frequency %		geometric mean of serum UA		
	WW	WM	MM	W	M	WW	WM	MM	WW	WM	MM	W	M	WW	WM	MM
rs18678	43	32	0	79	21	303	309	-	31	44	0	71	29	232	215	-
rs2240722	27	17	31	47	53	299	321	303	25	9	41	39	61	214	223	226
rs21155	53	22	0	85	15	312	290	-	51	24	0	84	16	218	229	-
rs2240720	12	9	54	22	78	283	321	308	19	4	52	28	72	217	221	224
rs4292327	75	0	0	100	0	305	-	-	75	0	0	100	0	222	-	-
rs8359	75	0	0	100	0	305	-	-	75	0	0	100	0	222	-	-
rs8361	75	0	0	100	0	305	-	-	75	0	0	100	0	222	-	-
rs7932775	74	1	0	99	1	305	327	-	75	0	0	100	0	222	-	-

	Hyperuricemic group - men (N = 86)								Hyperuricemic group - women (N = 14)							
significantly associated allelic variants	genotype distribution (N)			allele frequency %		geometric mean of serum UA			genotype distribution (N)			Allele frequency %		geometric mean of serum UA		
	WW	WM	MM	W	M	WW	WM	MM	WW	WM	MM	W	M	WW	WM	MM
rs18678	86	0	0	100	0	420	-	-	14	0	0	100	0	383	-	-
rs2240722	21	32	33	43	57	434	415	415	1	10	3	43	57	349	389	373
rs21155	83	3	0	98	2	423	326	-	14	0	0	100	0	383	-	-
rs2240720	22	27	37	41	59	422	448	399	1	7	6	32	68	349	386	384
rs4292327	44	36	6	72	28	430	404	440	5	8	1	64	36	404	374	350
rs8359	11	75	0	56	44	402	422	-	0	14	0	50	50	-	383	-
rs8361	11	75	0	56	44	402	422	-	0	14	0	50	50	-	383	-
rs7932775	67	10	9	84	16	417	421	435	12	2	0	93	7	381	392	-

	Primary gout/hyperuricemia subset - men (N = 43)								Primary gout/hyperuricemia subset - women (N = 3)							
significantly associated allelic variants	genotype distribution (N)			allele frequency %	geometric mean of serum UA				genotype distribution (N)			Allele frequency %		geometric mean of serum UA		
	WW	WM	MM	W	M	WW	WM	MM	WW	WM	MM	W	M	WW	WM	MM
rs18678	43	0	0	100	0	381	-	-	3	0	0	100	0	424	-	-
rs2240722	11	13	19	41	59	411	364	376	0	3	0	41	59	-	424	-
rs21155	40	3	0	97	3	385	326	-	3	0	0	97	3	424	-	-
rs2240720	12	8	23	37	63	391	433	359	0	1	2	37	63	-	449	413
rs4292327	20	22	1	72	28	387	373	428	1	2	0	72	28	498	392	-
rs8359	9	34	0	60	40	388	379	-	0	3	0	60	40	-	424	-
rs8361	9	34	0	60	40	388	379	-	0	3	0	60	40	-	424	-
rs7932775	30	10	3	81	19	366	421	411	1	2	0	81	19	498	392	-

## Discussion

Many genome-wide association studies have uncovered over 30 common sequence variants influencing serum UA concentrations and/or gout in several genes, mostly in *SLC2A9*, *SLC22A12* and *ABCG2*. However, the function characterization of the identified associated allelic variants for urate transporters URAT1 and GLUT9 was not studied. In this present study, we identified a total of 52 sequence variants (12 unpublished) in *SLC2A9* and *SLC22A12* although the non-synonymous allelic variants were found in *SLC2A9* only. We made the following new observations: (*a*) both urate uptake and expression of the found eight non-synonymous allelic variants of GLUT9 in the *Xenopus laevis* oocytes were not significantly changed in comparison with the native protein; (b) both isoforms of GLUT9 were expressed in the plasmatic membrane of *Xenopus laevis* oocytes and their uptake of urate was not statistically significantly different; (c) while some observed intronic variants in *SLC2A9* and *SLC22A12* did show differences in genotype distribution in hyperuricemic/gout patients, none are significantly associated with serum UA concentrations.

At present, 39 mutations in the *SLC22A12* gene (31 missense/nonsense, one splicing, two regulatory, three small deletions, one small insertion, and one gross deletion) have been described (HGMD Professional 2013.4). 33 of these 39 mutations cause renal hypouricemia type 1. Five URAT1 allelic variants have been described with a gout phenotype. It has been reported that among 69 Mexican patients with primary gout, 16 showed different URAT1 variants: p.R284G (CM072044), p.G290C (CM072045), p.Q297E (CM072047), p.I305S (CM072046) [32]; however, these base substitution could be related to silent polymorphisms instead of gain-of-function mutations. Variant p.G65W (rs12800450) was identified in African Americans [33] and a function study using ^14^C-urate transport assay in mammalian Chinese hamster ovary showed reduced urate transport and no genome-wide significant associations were observed for gout. The synonymous variant p.L437L (rs7932775) and intron polymorphism ss161109885 in URAT1 was described as having a joint additive effect of hyperuricemia in the Han Chinese. Individuals were carrying at least one change allele in ss 161109885 and homozygous changes at rs7932775 having a 5.88-fold increased risk of hyperuricemia in comparison with references sequence [34]. In our study, we did not find allelic variants in ss161109885. The genotype frequency of the rs7932775 variant in hyperuricemics significantly differed from the frequency in normouricemics (hyperuricemics having a greater proportion of the mutant allele). However, there was no statistically significant change in serum UA concentrations in mutant allele carriers. Collectively, no study has confirmed refuted URAT1 mutations in patient with gout and it is uncertain whether an enhanced tubular reabsorption of urate may cause hyperuricemia [35], [36].

Thus far, 17 mutations in the *SLC2A9* gene (11 missense/nonsense, three regulatory, one small insertion, one gross deletion and one gross insertion/duplication) have been described (HGMD Professional 2013.4). 12 of these 17 mutations cause renal hypouricemia type 2. Five mutations have been described with a gout phenotype: two missense variants p.V282I (rs16890979) [37], p.H294R (rs3733591) [38], [39] and three regulatory mutations [10], [40], [41]. The rs16890979 has been identified as being associated with serum UA concentrations, with a stronger association in women in the Framingham and Rotterdam cohorts [37] and in the island population of the Adriatic coast of Croatia [42]. Variant rs3733591 significantly contributes to the development of elevated serum UA concentrations and gout in the Han Chinese, Solomon Island and Japanese cohorts [25], [38], but not in the Eastern Polynesian, Western Polynesian cohorts and cohort of European descent [39], [42]. Intronic variant rs2240720 was not previously reported in associations with serum UA and/or gout. We have found a statistically significant difference in genotype distribution between the normo- and the hyperuricemic groups. However, there was no effect of the mutated allele on the serum UA concentrations. Previously reported associated studies in different ethnic groups [43] are thus inconsistent in the impact of genetic variants affecting serum UA and/or gout. This discrepancy is probably caused by linkage disequilibrium with the more strongly associated variants that include rs6855911, rs11942223 and rs16890979.

Recently, it has been reported that isoforms of GLUT9 variant 1 have unique intra tissue distribution in the human kidney. GLUT9 variant 1 is expressed in the basolateral membrane of kidney proximal tubules and this isoform is likely to be responsible for urate reabsorption and GLUT9 variant 2 is expressed at the apical membrane of collecting ducts [44]. In our study, both isoforms were expressed on the plasmatic membrane of *Xenopus laevis* oocytes and their uptake of urate was not significantly different. However, GLUT9 variant 1 exhibited stronger continuous immunostaining. Any GLUT9 allelic variants have not shown the absence of signal on the plasmatic membrane and/or in cytoplasm as we reported in allelic variants in URAT1 in patients with RHUC1 [18].

**Figure 4:**
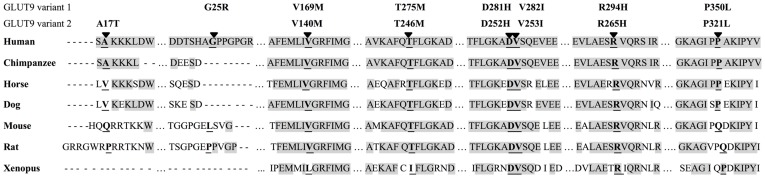
*SLC2A9* allelic variants. Alignment of the GLUT9 amino acids in the studied allelic variants with chimpanzee, horse, dog, mouse, rat and *Xenopus* paralogs.

An evolutionary analysis of GLUT9 paralogous sequences, including five mammalian species and *Xenopus tropicalis*, revealed a high conservation of allelic variants p.V169M, p.D281H, p.V282H, p.R294H and p.T275M. On the other hand, allelic variants with mutated positions p.A17T and p.G25R display almost no consensus. Both allelic variants are presented in the N terminus of GLUT9 which is responsible for protein localization to the basolateral or apical membrane of proximal tubular cells [45]. However, except for humans, UA is not reabsorbed in to the blood stream by the kidney, indicating the low evolutionary pressure to preserve the sequence of N terminus. Nonetheless, all other allelic variants analyzed in this paper showed strong evolutionary conservation across the taxa. Interestingly, the immunohistochemical and functional studies presented here showed normal membrane localization and UA transport in *Xenopus laevis* oocytes in all studied allelic variants. Thus, it seems that the analyzed mutations have no influence regarding UA influx. However, GLUT9 is also known as a transporter of hexoses. We concluded that evolutionary conservation of studied allelic variants reflects rather hexose than UA transport, Figure 4.

Serum UA concentration is a complex phenotype influenced by both genetic and environmental factors as well as their interactions. In the largest studies, sequence variants in *SLC2A9* and *SLC22A12* have demonstrated a strong effect on urate transport. However, the functions of these transporters seem not only to be solely dependent on their expression levels or structures but are also affected by the concentration of counter anions and by the sex hormones which appear to regulate urate transporters [46].

Our study has several strengths. First, we explored the sequencing analysis of *SLC22A12* and *SLC2A9* in a general Czech population of European descent with detailed characterization that allowed us to answer our research questions and that could be generalized to a larger population. Second, we controlled for several potential confounders, such as the types of medications that may influence serum UA concentrations. Third, we used a complex approach; not only did we perform an associations study; we also included a function and immunohistochemical characterization of non-synonymous allelic variants of both isoforms of GLUT9. Then again, some limitations of this study should also be acknowledged. First, the size of the studied groups may not be sufficiently large for such detailed analysis and some possibly associated variants may have stayed undetected. Second, the number of the frequent genetic variants of genes encoding the urate transporters was limited into transcription regions and exon-intron boundaries. Third, URAT1 and GLUT9 activity may be different in *Xenopus laevis* oocytes compared to humans. The proteins being evaluated are expressed in the renal epithelial cells which will traffic proteins in a different way from the non-polarized oocyte.

To our best of knowledge, this is the first study of the impact of the non-synonymous allelic variants on the function of *GLUT9* except for patients suffering from renal hypouricemia type 2. Our results did not show the influence of the identified non-synonymous allelic variants on urate uptake, subcellular localization and serum uric acid concentrations and/or gout. Given the heterogeneity evident in risk conferred for hyperuricemia at *SLC2A9* and *SLC22A12* in different populations studied so far, further investigation of these genes is warranted. However, with the accelerated identification of association variants, it will be increasingly important to experimentally assess whether these variants that cause amino acid substitution are indeed responsible for hyperuricemia and gout.

## Supporting Information

Table S1 **List of all identified variants in 250 Czech subjects in coding region and intron-exon boundaries in** ***SLC2A9*** **and** ***SLC22A12*** **genes (their position, function, genotype distribution and allelic frequency).** Reference sequence: *SLC2A9* NM_001001290.1, *NP_001001290.1 and NP_064425.2, *SLC22A12* NM_001276326.1 and NP_001263255.1.
(DOC)

Table S2 **Primer sequences (5′-3′) and PCR conditions used for amplification of promoter and coding regions of the** ***SLC2A9*** **and** ***SLC22A12*** **genes.** Primers have overhangs added - forward primers T7 sequence 5′-AAT ACG ACT CAC TAT AG-3′, reverse primers RP sequence 5′-GAA ACA GCT ATG ACC ATG-3′ (exception – *SLC2A9* exon 3 variant 2 is without overhangs).
(DOCX)
