# Supplementary material for: Complex Analysis of Urate Transporters SLC2A9, SLC22A12 and Functional Characterization of Non-Synonymous Allelic Variants of GLUT9 in the Czech Population: No Evidence of Effect on Hyperuricemia and Gout
Source: PLoS One. 2014 Sep 30;9(9):e107902. doi: 10.1371/journal.pone.0107902 (PMC4182324; doi:10.1371/journal.pone.0107902)
Supplement: Table S1 — List of all identified variants in 250 Czech subjects in coding region and intron-exon boundaries in SLC2A9 and SLC22A12 genes (their position, function, genotype distribution and allelic frequency). Reference sequence: SLC2A9 NM_001001290.1, *NP_001001290.1 and NP_064425.2, SLC22A12 NM_001276326.1 and NP_001263255.1. (DOC) [file pone.0107902.s001.doc]

**Table S2**. Allele frequency of the studied variants and the p-values of the Fisher exact test and goodness-of-fit test in the association models.

| sequence variant | Normouricemics  (N = 150) | | Hyperuricemics  (N = 100) | | Primary hyperuricemia/  gout (N = 46) | | Normouricemics vs. hyperuricemics | Normouricemics vs. pr. hyperuricemia/  gout patients | Normouricemics - regression model |
| --- | --- | --- | --- | --- | --- | --- | --- | --- | --- |
|  | W | M | W | M | W | M | p-value  Fisher exact test | p-value  Fisher exact test | p-value goodness-of-fit test |
| rs6449237 | 94% | 6% | 93% | 7% | 95% | 5% | 0.7527 | 0.6616 | 0.9749 |
| rs6820230.SNP | 94% | 6% | 93% | 7% | 95% | 5% | 0.7527 | 0.6616 | 0.9749 |
| rs61256984.DELETION | 94% | 6% | 92% | 8% | 92% | 8% | 0.2504 | 0.0751 | 0.9749 |
| rs18678 | 75% | 25% | 100% | 0% | 100% | 0% | 0.0000 | 0.0000 | 0.2364 |
| rs18680 | 100% | 0% | 100% | 0% | 99% | 1% | 0.4000 | 0.2347 | monomorphic |
| rs18687 | 100% | 0% | 100% | 0% | 100% | 0% | 1.0000 | 1.0000 | 0.9888 |
| rs2276961 | 47% | 53% | 46% | 54% | 47% | 53% | 0.9401 | 0.8895 | 0.1621 |
| rs112581525 | 100% | 0% | 100% | 0% | 100% | 0% | 1.0000 | 1.0000 | 0.9318 |
| rs138627925 | 100% | 0% | 100% | 0% | 100% | 0% | 1.0000 | 1.0000 | 0.4131 |
| rs2276962 | 98% | 2% | 99% | 1% | 99% | 1% | 0.3645 | 0.9175 | 0.3101 |
| rs2276963 | 98% | 2% | 100% | 0% | 100% | 0% | 0.1314 | 0.5380 | 0.3101 |
| rs21082 | 99% | 1% | 100% | 0% | 100% | 0% | 0.1522 | 0.5747 | 0.0577 |
| rs21099 | 99% | 1% | 100% | 0% | 100% | 0% | 0.1522 | 0.5747 | 0.0577 |
| rs2240722 | 43% | 57% | 44% | 56% | 41% | 59% | 0.0001 | 0.0456 | 0.2824 |
| rs21155 | 85% | 15% | 99% | 1% | 96% | 4% | 0.0000 | 0.0003 | 0.6794 |
| rs21278 | 96% | 4% | 100% | 0% | 100% | 0% | 0.0020 | 0.0723 | 0.2826 |
| rs21300 | 96% | 4% | 100% | 0% | 100% | 0% | 0.0021 | 0.0412 | 0.6599 |
| rs2240721 | 36% | 64% | 41% | 59% | 37% | 63% | 0.3898 | 0.9858 | 0.2367 |
| rs2240720 | 25% | 75% | 41% | 59% | 36% | 64% | 0.0000 | 0.0742 | 0.2074 |
| rs28592748 | 23% | 77% | 18% | 82% | 25% | 75% | 0.0415 | 0.0488 | 0.3378 |
| rs13113918 | 23% | 77% | 17% | 83% | 22% | 78% | 0.0575 | 0.0951 | 0.4827 |
| rs10939650 | 23% | 77% | 20% | 80% | 24% | 76% | 0.3446 | 0.1201 | 0.4106 |
| rs43466 | 99% | 1% | 100% | 0% | 100% | 0% | 0.2771 | 1.0000 | 0.7060 |
| rs16891971 | 100% | 0% | 99% | 1% | 99% | 1% | 0.1590 | 0.2347 | monomorphic |
| rs3733589 | 98% | 2% | 98% | 2% | 99% | 1% | 1.0000 | 0.9175 | 0.4372 |
| rs144196049 | 100% | 0% | 100% | 0% | 99% | 1% | 0.4000 | 0.2347 | monomorphic |
| rs3733590 | 98% | 2% | 97% | 3% | 97% | 3% | 0.3009 | 0.4917 | 0.0204 |
| rs13125646 | 21% | 79% | 17% | 83% | 24% | 76% | 0.0864 | 0.0297 | 0.7766 |
| rs13115193 | 48% | 52% | 52% | 48% | 57% | 43% | 0.4204 | 0.4963 | 0.6975 |
| rs4292327 | 100% | 0% | 71% | 29% | 70% | 30% | 0.0000 | 0.0000 | monomorphic |
| rs112404957 | 99% | 1% | 99% | 1% | 100% | 0% | 0.5245 | 1.0000 | 0.7623 |
| rs73225891 | 98% | 2% | 95% | 5% | 96% | 4% | 0.0889 | 0.4023 | 0.1115 |
| rs16890979 | 79% | 21% | 85% | 15% | 86% | 14% | 0.1537 | 0.3099 | 0.1722 |
| rs3733591 | 80% | 20% | 85% | 15% | 86% | 14% | 0.1566 | 0.5675 | 0.2660 |
| rs6823877 | 37% | 63% | 44% | 56% | 45% | 55% | 0.0890 | 0.2631 | 0.2566 |
| rs2280205 | 49% | 51% | 46% | 54% | 47% | 53% | 0.3159 | 0.1247 | 0.6981 |
| rs2280204 | 84% | 16% | 83% | 17% | 84% | 16% | 0.9606 | 0.8239 | 0.1829 |
| rs114361719 | 100% | 0% | 100% | 0% | 99% | 1% | 0.4000 | 0.2347 | monomorphic |
| rs149641 | 99% | 1% | 100% | 0% | 100% | 0% | 0.2771 | 1.0000 | 0.4224 |
| rs144428359 | 100% | 0% | 100% | 0% | 99% | 1% | 0.4000 | 0.2347 | monomorphic |
| rs3825017 | 100% | 0% | 100% | 0% | 100% | 0% | 1.0000 | 1.0000 | 0.0519 |
| rs3825016 | 34% | 66% | 40% | 60% | 37% | 63% | 0.1606 | 0.1537 | 0.8727 |
| rs11231825 | 36% | 64% | 36% | 64% | 34% | 66% | 0.5766 | 0.1186 | 0.9854 |
| rs8087 | 100% | 0% | 99% | 1% | 100% | 0% | 0.4000 | 0.2347 | monomorphic |
| rs8359 | 100% | 0% | 55% | 45% | 57% | 43% | 0.0000 | 0.0000 | monomorphic |
| rs8361 | 100% | 0% | 55% | 45% | 57% | 43% | 0.0000 | 0.0000 | monomorphic |
| rs7932775.SNP | 100% | 0% | 85% | 15% | 79% | 21% | 0.0000 | 0.0000 | 0.6377 |
| rs11231837 | 83% | 17% | 76% | 24% | 78% | 22% | 0.0007 | 0.0193 | 0.9344 |
| rs138485972 | 100% | 0% | 99% | 1% | 100% | 0% | 0.4000 | monomorphic | monomorphic |
| rs11602903 | 35% | 65% | 37% | 63% | 36% | 64% | 0.5098 | 0.2203 | 0.8149 |
| rs524023 | 65% | 35% | 63% | 37% | 64% | 36% | 0.5098 | 0.2203 | 0.8149 |
| rs9734313 | 35% | 65% | 38% | 62% | 37% | 63% | 0.5332 | 0.3236 | 0.814 |
