# Supplementary material for: Complex Analysis of Urate Transporters SLC2A9, SLC22A12 and Functional Characterization of Non-Synonymous Allelic Variants of GLUT9 in the Czech Population: No Evidence of Effect on Hyperuricemia and Gout
Source: PLoS One. 2014 Sep 30;9(9):e107902. doi: 10.1371/journal.pone.0107902 (PMC4182324; doi:10.1371/journal.pone.0107902)
Supplement: Table S2 — Primer sequences (5′-3′) and PCR conditions used for amplification of promoter and coding regions of the SLC2A9 and SLC22A12 genes. Primers have overhangs added - forward primers T7 sequence 5′-AAT ACG ACT CAC TAT AG-3′, reverse primers RP sequence 5′-GAA ACA GCT ATG ACC ATG-3′ (exception – SLC2A9 exon 3 variant 2 is without overhangs). (DOCX) [file pone.0107902.s002.docx]

**Table S2**. Primer sequences (5´-3´) and PCR conditions used for amplification of promoter and coding regions of the *SLC2A9* and *SLC22A12* genes. Primers have overhangs added - forward primers T7 sequence 5´-AAT ACG ACT CAC TAT AG-3´, reverse primers RP sequence 5´-GAA ACA GCT ATG ACC ATG-3´ (exception – *SLC2A9* exon 3 variant 2 is without overhangs).

| gene/variant | exon | size of product, bp | primer sequences (forward) | primer sequences (reverse) | annealing temperature, °C |
| --- | --- | --- | --- | --- | --- |
| *SLC2A9* var.1 | 1 | 504 | TTTGGACTGTTTCATTGCTGT | CCCAGATCCCCCAGCTACAC | 58 |
|  | 2 | 424 | CTTGCGTCTCTTCTCTCTGTC | GAGTGGGGGCTAATCTCTGTC | 58 |
|  | 3 | 477 | GCCAATGAGATAAAGCTCAAA | CCTTTGGGCATTTGAATCTCT | 62 |
|  | 4 | 420 | GTGCCTGAGTCTTGCTCTACT | AGAGTTCCAGCATCCTTGGTC | 62 |
|  | 5 | 445 | TTGTGCTAAGTGCTGGGAAA | TAAGAGGCTTGAATACCAGAA | 62 |
|  | 6 | 454 | AGTTCCTTCCCTCAGCCTTTG | CCCTATGATACCCAGCCCAGT | 62 |
|  | 7 | 457 | TAAGGTGTGTGGCTAGGAGGT | ACAGCATAGAGTTTGTGGCAAT | 62 |
|  | 8 | 346 | CACCTTGGGACTCTGAGTTTA | TGTGCCTTAAAGGACCTTATG | 62 |
|  | 9 | 327 | GTCTCAGGGTCAAAGCCGGTT | CCCCAAAACGATGAAGCCAGA | 66 |
|  | 10 | 353 | GGGGACATCAAGTGACCTGTC | ATCCCCAGTCAGGCTTTGCTT | 58 |
|  | 11 | 361 | TGGGGGGGTTCTAAAGTGTG | TCCAGTTGAGAGGAGAGAGATC | 66 |
|  | 12 | 638 | TGGTGTTTTGTCTAGGAGTAT | ATAGAAGAACGCTGGGTCTAC | 68 |
| *SLC2A9* var.2 | 3 | 387 | CTGTGTCCTATCCTGGCATCC | CTGCTGCTCACTCTTTTCTCT | 61,4 |
| *SLC22A12* | promotor | 317 | GTGGGGCTCTGCAGGAGGCATAGA | CTGGGGCAGCTGTGCAAGTCTCTG | 66 |
|  | 1 | 727 | CCTCACGCGGCCTCAGGGCCCAGTT | GGGTCCCTCCCAGGACTGGACCTTT | 64 |
|  | 2 | 416 | CCCTCACTGTTCCACAGGGTCTTGCTCT | CCAGCAAGTAGGGCGCTTTCTAGACTTG | 66 |
|  | 3-4 | 572 | CATAGGGTGGGCTCTAGGTGTTCCAGAG | GGAGAGTGGGCAGGATCTCCTCTGAGGA | 64 |
|  | 5-6 | 565 | GCCAGGCACTGGGGGCCACAGGCAAT | CCGTGTGCCAGCGAGAGCCCCGATT | 66 |
|  | 7 | 547 | GCCCCCACCGCCCATTGTTCC | CTCCGGCAGCCTCCTTCCTACCAG | 66 |
|  | 8 | 298 | GCTGAAGGGAGCCCTCATCTGATCTTGG | TAGGTCTGGGAGAAGCCAGTCCTGCCTG | 66 |
|  | 9 | 419 | GCCTGGCCCGGCGTGCAGGATCAA | GCCTCTGCTTCCGCCTCTGTCAA | 64 |
|  | 10 | 397 | CTGGGCCCCCGAGAGCA | GGGGCCCCTTGGACTGTGAG | 66 |
